# *Dendrobium officinale* Flower Extraction Mitigates Alcohol-Induced Liver Injury in Mice: Role of Antisteatosis, Antioxidative, and Anti-Inflammatory

**DOI:** 10.1155/2020/1421853

**Published:** 2020-10-14

**Authors:** Yu-Lin Wu, Si-Han Huang, Chun-Mei He, Bo Qiu, Jing-Jing Liu, Jia Li, Ying Lin, Sheng-Lu Yu, Hong-Feng Wang, Gui-Fang Zhang

**Affiliations:** ^1^Guangdong Provincial Key Laboratory of New Drug Development and Research of Chinese Medicine, Mathematical Engineering Academy of Chinese Medicine, Guangzhou University of Chinese Medicine, Guangzhou 510000, China; ^2^Medical College of Jiaying University, Jiaying University, Meizhou 514000, China; ^3^Guangdong Provincial Key Laboratory of Silviculture, Protection and Utilization, Guangdong Academy of Forestry, Guangzhou 510000, China; ^4^School of Pharmaceutical Sciences, Guangzhou University of Chinese Medicine, Guangzhou 510000, China; ^5^Liannan Yao Autonomous County Xinshengtang Biological Technology Co., Ltd., Qingyuan 511500, China

## Abstract

The study aimed to evaluate the protective effect of *Dendrobium officinale* flower extraction (DOFE) on alcohol-induced liver injury and its probable mechanisms in mice. The chemical composition of DOFE was performed via UPLC/MS. Male Kunming mice were used to establish alcohol-induced liver injury models by oral gavage of 56% alcohol. Results showed that DOFE dramatically attenuated the increased serum levels of alanine aminotransferase (ALT), aspartate aminotransferase (AST), total cholesterol (TC), and triacylglycerol (TG). Meanwhile, hematoxylin and eosin and Oil Red O staining showed that DOFE attenuated degeneration, inflammatory infiltration, and lipid droplet accumulation. DOFE was also found to suppress the activity of malonaldehyde (MDA) and enhanced the level of glutathione (GSH) and the activities of superoxide dismutase (SOD), glutathione peroxidase (GSH-Px), and catalase (CAT) in the liver. The protection of DOFE against oxidative stress was associated with the downregulation of hepatic cytochrome P450 2E1 (CYP2E1) and upregulation of nuclear factor erythroid 2-related factor 2 (Nrf2), heme oxygenase-1 (HO-1), and NAD(P)H quinone oxidoreductase l (NQO1). Additionally, DOFE suppressed inflammation via downregulating Toll-like receptor-4 (TLR-4) and nuclear factor kappa-B P65 (NF-*κ*B P65). Thus, DOFE exhibited a significant protective effect against alcohol-induced liver injury through its antisteatosis, antioxidative, and anti-inflammatory effect.

## 1. Introduction

Excess consumption of alcohol is a common cause of alcohol-induced liver disease (ALD) [1]. Each year, alcohol consumption contributes to about 3 million deaths according to an estimate by the World Health Organization. And the harmful use of alcohol is responsible for 5.1% of the global burden of disease [2]. Usually, ALD begins with simple hepatic steatosis and then develops to alcoholic steatohepatitis, fibrosis, and cirrhosis [3].

Previous studies have demonstrated that alcoholic liver injury is associated with steatosis, oxidative stress, and inflammation [4–6]. Normally, 90% of the alcohol is eliminated primarily in the liver [7]. Firstly, alcohol is converted into acetaldehyde in the presence of catalyst alcohol dehydrogenase (ADH) and cytochrome P450 2E1 (CYP2E1). Then, the acetaldehyde is oxidized to acetic acid with the involvement of acetaldehyde dehydrogenase (ALDH2). Finally, the acetic acid is metabolized to carbon dioxide by the citric acid cycle [8]. However, the ability of liver to metabolize alcohol is limitary, and the alcohol-induced hepatotoxicity is mainly mediated through its conversion to acetaldehyde [9, 10]. On the one hand, the acetaldehyde can induce oxidative stress, decrease the amount of glutathione, and increase the production of reactive oxygen species (ROS) [8]. On the other hand, excess ROS induces the accumulation of lipid in hepatocytes, which leads to liver steatosis. In addition, overconsumption of alcohol activates Kupffer cells to generate a large amount of nuclear factor kappa-B which promotes the release of tumor necrosis factor-*α* (TNF-*α*) and other inflammatory cytokines [11, 12]. In another way, alcohol oxidation results in generation of nicotinamide adenine dinucleotide (NADH), which inhibits fatty acid oxidation but promotes fatty acid and triglyceride synthesis [3]. Therefore, preventing oxidative stress and inflammation would be an effective method in delaying the pathogenesis of alcohol-induced liver injury.

Clinically, the general treatment drugs of alcohol-induced liver injury include glucocorticoid, a phosphodiesterase inhibitor, and polyene phosphatidylcholine [13]. Although these drugs can reduce liver damage, they remain suboptimal with a marginal short-term survival benefit. Besides, they can increase the risk of infection and complications in patients. Therefore, it is greatly significant to investigate the pathogenesis of alcoholic liver disease and develop effective treatment medicine in the treatment and prevention of alcoholic liver disease. Recently, herbal medicines have attracted increasing attention in the treatment of alcoholic liver injury, due to the wide availabilities, multitarget actions, and low side effects [14–16]. Chinese herbal medicines of saponins from the leaves of *Panax notoginseng*, *Camellia nitidissima,* and phenol have been used to improve the alcohol-induced liver injury [6, 17, 18].

*Dendrobium officinale* Kimura et Migo *(D. officinale)*, original food material and medical plant, is an important traditional Chinese herbal medicine [19]. The flowers of *D. officinale* have been traditionally used for tea making and cooking. In 2018, the Chinese authority approved the *D. officinale* flowers as a new food ingredient [20, 21]. The flowers of *D. officinale* have been shown that it would be a source containing a high amount of antioxidant phenolic components [22]. Besides, the flowers of *D. officinale* contained essential and nonessential amino acids [23], polysaccharides, and volatile components [24, 25]. Pharmaceutical studies revealed that *D. officinale* flowers exhibited DPPH radical scavenging activity and antihypertensive effect [21]. The animal studies demonstrated that *D. officinale* flowers protected against liver injury caused by an overactive thyroid axis [26], high glucose, and high-fat compound alcohol-induced hypertensive [27]. However, the effect of *D. officinale* flower extraction (DOFE) against alcohol-induced liver injury remains unknown. Therefore, we speculate that *D. officinale* flowers can protect against alcohol-induced liver injury.

This study aimed to systematically investigate whether DOFE exerted protective effects on alcohol-induced liver injury related to antisteatosis, antioxidative, and anti-inflammatory effects.

## 2. Materials and Methods

### 2.1. Materials and Chemicals

The dried flowers of *D. officinale* were provided by Guangdong Academy of Forestry (Guangzhou, China). Beijing Sorghum Spirit (56% vol) was purchased from Beijing Jingying Wine Co. Ltd. (Beijing, China), and bifendate (98%, 73536-69-3) was purchased from Shanghai Macklin Biochemical co. LTD (Shanghai, China). ALT (C009-2-1) detection kit, AST (C010-2-1) detection kit, hepatic lipid levels of TC (A111-1-1) detection kit, TG (A110-1-1) detection kit, GSH (A006-2) assay kit, GSH-Px (A005) assay kit, CAT (A007-1-1) assay kit, SOD (A001-1) assay kit, and MDA (A003-1) assay kit were obtained from Nanjing Jiancheng Bioengineering Institute (Nanjing, China). The enzyme-linked immunosorbent assay (ELISA) kits TNF-*α* (ml002095), IL-1*β* (ml063132), and IL-6 (ml063159) were purchased from Shanghai Enzyme Link Biotechnology Co. Ltd. (Shanghai, China). BCA kit (BB-3401) was purchased from Shanghai Beibo Biotechnology Co. Ltd. (Shanghai, China). A nuclear protein and cytoplasmic protein extraction kit (KGP150/KGP1100) was purchased from Nanjing Kaiji Biotechnology Development Co. Ltd. (Nanjing, China). CYP2E1 (DF6883), Nrf2 (AF0639), HO-1 (AF5393), NQO1 (DF6437), TLR4 (AF7017), NF-*κ*B P65 (AF5006), Anti-Histone H3 antibodies (AF0863), and secondary antibodies (110191) were purchased from Affinity Biosciences (Inc., USA). Antibody against *β*-actin (ab8227) was purchased from Abcam (Cambridge, MA, USA). Reagents for Western Blotting were purchased from Bio-Rad (California Hercules, USA). Other materials in the experiments were analytically pure and obtained from local suppliers.

### 2.2. Extraction of DOFE

The dried *Dendrobium officinale* flowers were soaked 2 h in advance, refluxed thrice with 3-fold volumes of water for 1 h, filtered, and then concentrated by reducing pressure. The concentrated extract after filtration in the vacuum was freeze-dried. In this way, the *Dendrobium officinale* flowers extract (DOFE) was obtained and stored at 4°C.

### 2.3. Chemical Composition Analysis

The qualitative analysis of components in DOFE was carried out by a UPLC/qExactive-MS method. The analysis was performed on a UPLC instrument coupled with a Q Exactive spectrometer (Thermo Fisher, New York, United States) via an ESI ion source. Two hundred milligrams of DOFE was dissolved in 1 mL 80% methanol (HPLC grade) and then centrifuged at 20,000 × g, 4°C for 10 min to collect the supernatant. The supernatant was filtered through a filter (0.22 *μ*m pore size) for analysis.

The sample was separated on a common RP-C18 column (2.1 mm × 150 mm, 1.8 *μ*m; Welch). The column temperature was maintained at 35°C. The mobile phase consisted of water containing 0.1% (v/v) formic acid (A) and acetonitrile containing 0.1% (v/v) formic acid (B) at a flow rate of 0.3 mL/min. The gradient elution program was set as follows: 0–1 min, 98% A; 1–5 min, 98–80% A; 5–10 min, 80–50% A; 10–15 min, 50–20% A; 15–20 min, 20–5% A; 20–25 min, 5% A; 25–26 min, 5–98% A; 26–30 min, 98% A. The injection volume was 5 *μ*L. High-purity nitrogen (N_2_) and high-purity argon (Ar) were used as collision gas and desolvation gas, respectively. The capillary and auxiliary gas heater temperatures were 300°C and 350°C, respectively. The MS full scan range was 150–2000 m/z. All data collected were acquired and processed by the CD2.1 software (Thermo Fisher) and then retrieved and compared in the database (mzCloud, mzVault, ChemSpider).

### 2.4. Animal Experiments

The male Kunming mice, weighing 20 ± 2 g and aging 8 weeks old, were provided by the Laboratory Animal Center of Guangzhou University of Chinese Medicine (Guangzhou, China). All performance accorded with the Animal Experimental Ethics Committee of Guangzhou University of Chinese Medicine (Guangzhou, China, 20190531003).

The mice were adaptively raised for 7 days with random water and diet with a 12 h light/dark cycle under the controlled temperature (22 ± 2°C) and relative humidity (55% ± 5%). All mice were randomly assigned to the following six groups (*n* = 10): NC, MO, positive control group (PC, 150 mg/kg bifendate), a low dose of DOFE group (50 mg/kg), a moderate dose of DOFE group (100 mg/kg), and a high dose of DOFE group (200 mg/kg). Mice in the DOFE-treated groups and the PC group were orally given a corresponding dose of DOFE or bifendate. In contrast, in the NC and MO groups, mice were administered equal volumes of distilled water by gastric gavage. Two hours after each administration, all mice were intragastrically given 56% alcohol (10 mL/kg), except those in the NC group. The whole gavage procedure lasted for consecutive 10 days. On the last day, all mice fasted for 16 h. Blood subsequently was collected while fasting mice were anesthetized. Then, the mice were sacrificed, and the liver was carefully removed, weighed. The liver index was calculated according to the following calculation formula: The liver index = mice liver weight/mice weight × 100%. The hepatic tissues were harvested and washed with cold saline. Some tissues were fixed in 4% paraformaldehyde for histological evaluation and others were stored at−80°C for further analysis.

### 2.5. Histopathologic Evaluation

The liver tissues of those fixed in 4% paraformaldehyde were dehydrated and then embedded in paraffin. Then, samples were sectioned at 5 *µ*m thickness and finally dyed with hematoxylin and eosin (H&E) routinely. Liver histopathologic changes were captured with an optical microscope at 200x magnification (E100, Nikon Corporation).

### 2.6. Oil Red O Staining

The frozen liver tissues were embedded in a frozen tissue matrix and then sectioned at 8 *µ*m thickness. Finally, the sections were stained with Oil Red O (Sigma-Aldrich). The hepatic steatosis was observed from the Oil Red O staining by light microscopy (E100, Nikon Corporation).

### 2.7. Serum Biochemical Assays

The blood samples were kept at room temperature for 2 h and then centrifuged at 3000 rpm for 10 min at 4°C. Then, the serum was obtained, and the activities of ALT, AST, TC, and TG in the mice serum were determined by using commercials kits.

### 2.8. Liver Biochemical Assays

In brief, the liver tissues were kept in ice-cold saline (1 : 9, w/v) and then homogenized. The homogenate was centrifuged at 3000 rpm for 10 min at 4°C to separate the supernatant solution that was used to measure GSH, MDA levels, and GSH-PX, SOD, and CAT activities by commercial kits.

### 2.9. Enzyme-Linked Immunosorbent Assays (ELISA)

The liver tissues were thawed and then homogenized in cold PBS (1 : 9, w/v). After that, liver homogenates were centrifuged at 3000 rpm for 20 min. The assays of TNF-*α*, IL-1*β*, and IL-6 were performed by ELISA kits.

### 2.10. Reverse Transcription-Quantitative Polymerase Chain Reaction (RT-PCR)

The total RNA was extracted from liver tissue samples with Trizol reagent and reverse transcribed into cDNA. RNA quantities were measured at 260 nm to 280 nm. And the purity of RNA was identified between 1.8 and 2.0. The expressions of CYP2E1, HO-1, NQO1, and glyceraldehyde-3-phosphate dehydrogenase (GAPDH) in each sample were determined using HiScript® II Q RT SuperMix (+gDNA wiper) and ChamQ™ SYBR® qPCR Master Mix Kit. The mouse primer sequences were designed by using online primer design software (Sangon Biotech Co., Ltd., Shanghai), shown in Table 1. The amplification was performed, and the fluorescence intensity of the samples was determined by the monitor in real time. The gene expression of relative quantification was calculated using the 2^−ΔΔCq^ method using GAPDH as an internal reference.

### 2.11. Western Blotting Analysis

The liver tissue samples were homogenized in RIPA buffer with 1% of protease inhibitor cocktail and centrifuged at 14000 g for 10 min at 4°C. Then, the supernatant was removed and obtained total proteins. Besides, the protein of cytoplasmic and nucleus was extracted by the extraction kit (Thermo). And to determine the protein concentrations, the BCA Protein Assay Kit (Nanjing, Jiancheng) was used. The equivalent proteins of liver samples were separated by SDS-PAGE and then transferred to a polyvinylidene difluoride (PVDF) membrane. After that, the membranes with 5% skimmed milk were blocked under the room temperature for 1 h and then incubated with primary antibody of CYP2E1 (1 : 2000), Nrf2 (1 : 1000), HO-1 (1 : 1000), NQO1 (1 : 1000), TLR4 (1 : 3000), NF-*κ*B P65 (1 : 1000), and *β*-actin (1 : 3000) at 4°C overnight, respectively. Next, each membrane with an HRP-conjugated secondary antibody was incubated under the room temperature for 2 h. Subsequently, the membranes with ECL reagent were visualized and detected by Western Blotting Detection System. The density of each band was analyzed using ImageJ software.

### 2.12. Statistical Analysis

All data were expressed as mean ± SD, and statistical analysis was performed with a one-way analysis of variance (ANOVA) using SPSS software 23.0. The figures were conducted using GraphPad Prism 8.0.1. The difference was considered statistically significant at the level of *p* < 0.05.

## 3. Results

### 3.1. Chemical Composition of DOFE

**Figure 1:**
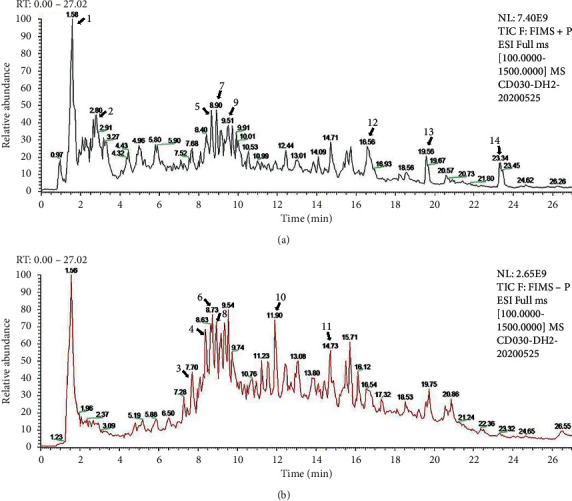
Chemical composition of DOFE. (a) Positive mode. (b) Negative mode.

The total ion chromatogram of DOFE is shown in Figure 1, and the characterization of the compounds is presented in Table 2.

### 3.2. Effect of DOFE on Alcohol-Induced Liver Injury

**Figure 2:**
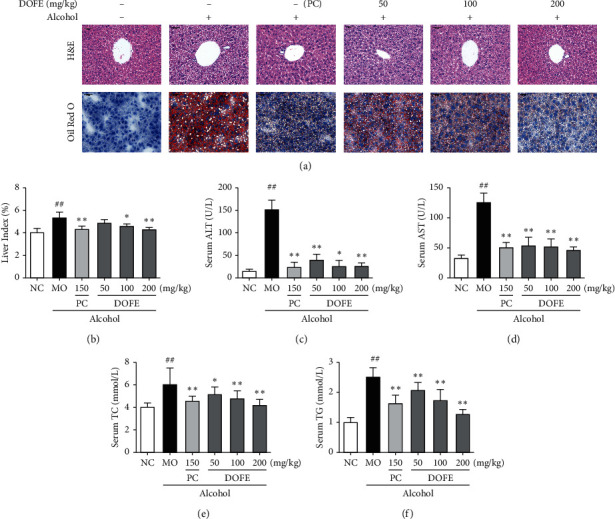
Effect of DOFE on alcohol-induced liver steatosis and injury. (a) Histological examination of liver sections stained with H&E and hepatic steatosis of liver sections stained with Oil Red (O) (200x magnification). (b) Liver index. Serum level of (c) ALT, (d) AST, (e) TC, and (f) TG. All data are expressed as mean ± SD (*n* = 8). #*p* < 0.05 and ##*p* < 0.01 vs. normal control group; ^*∗*^*p* < 0.05 and ^*∗∗*^*p* < 0.01 vs. model group.

To evaluate the effects of DOFE on alcohol-induced liver injury, liver index, hepatic histopathological changes, hepatic steatosis, and plasma markers were examined in alcohol-induced liver injury mice (Figure 2).

Hepatic cells had prominent nucleolus, complete pattern cytoplasm, and portal vein in the normal control (NC) group. However, the major histopathological change in the liver from the model (MO) group was lipid droplet accumulation. As shown in the cytosolic compartment and hepatic steatosis of liver sections stained with Oil Red O, the lipid vesicles were observed. However, fatty vesicles in either bifendate or DOFE treatment groups were much fewer than those of the MO group (Figure 2(a)). Compared with the NC group, the liver index in the MO group was remarkably increased by 32.6% (*p* < 0.01). However, DOFE (100 and 200 mg/kg) treatment significantly reversed the increase (*p* < 0.05, *p* < 0.01), while there was no significant difference between the MO group and DOFE (50 mg/kg) treatment group (Figure 2(b)).

The serum levels of alanine aminotransferase (ALT) and aspartate aminotransferase (AST) in the MO group were significantly increased by 896.7% (15.4 ± 3.8 vs 153.0 ± 19.7) and 282.0% (33.4 ± 4.2 vs 127.4 ± 13.4), compared to the NC group. In contrast, these elevations were changed by the treatment with either bifendate or DOFE (all, *p* < 0.01). The results indicated the hepatoprotective effects of DOFE on alcoholic liver injury. Surprisingly, DOFE (50 mg/kg) treatment decreased the serum ALT level in comparison to the MO group (*p* < 0.01) (Figures 2(c) and 2(d)). In addition, alcohol exposure also dramatically increased serum total cholesterol (TC) and triacylglycerol (TG) contents by 54.0% and 145.4% compared with the NC group (both, *p* < 0.01), and these elevations were significantly reversed by either bifendate or DOFE (100 and 200 mg/kg) treatments (all, *p* < 0.01) (Figures 2(e) and 2(f)). These results demonstrated that DOFE could effectively protect against alcohol-induced liver injury.

### 3.3. Effect of DOFE on Alcohol-Induced Oxidative Stress

**Figure 3:**
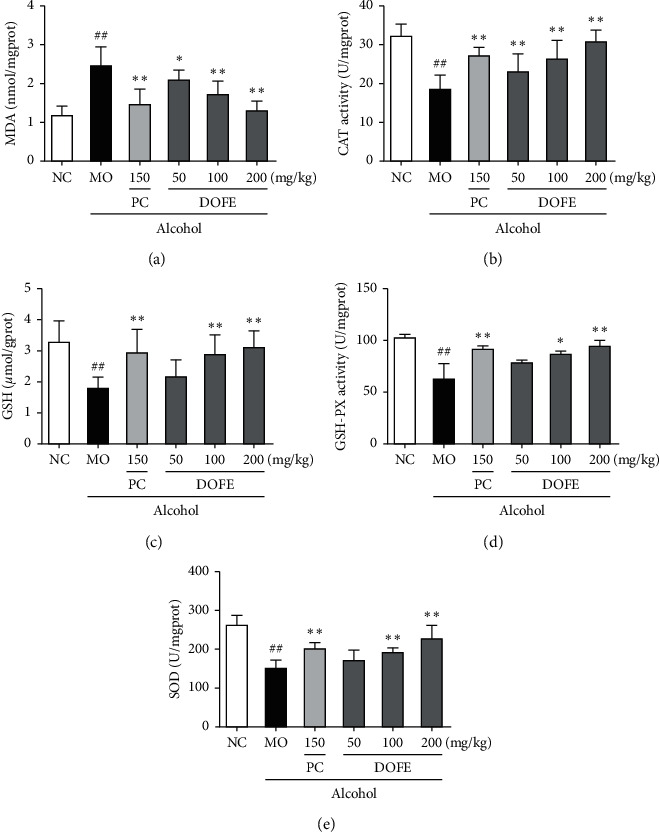
Effect of DOFE on alcohol-induced oxidative stress. (a) MDA; (b) CAT; (c) GSH; (d) GSH-PX; (e) SOD. All data are expressed as mean ± SD (*n* = 8). #*p* < 0.05 and ##*p* < 0.01 vs. normal control group; ^*∗*^*p* < 0.05 and ^*∗∗*^*p* < 0.01 vs. model group.

To evaluate the effects of DOFE on alcohol-induced oxidative stress, antioxidant enzyme and lipid peroxidation activities were examined (Figure 3). Alcohol induced the remarkable increase of malonaldehyde (MDA) production by 106.9% (1.2 ± 0.2 vs 2.5 ± 0.4, *p* < 0.01). Consistently, the treatment of DOFE (100 and 200 mg/kg) significantly decreased the alcohol-induced hepatic lipid peroxidation (both, *p* < 0.01). Conversely, the activities of catalase (CAT), superoxide dismutase (SOD), glutathione peroxidase (GSH-Px), and the levels of glutathione (GSH) were significantly decreased in the alcohol treatment group compared with the NC group (all, *p* < 0.01). The depletion of these antioxidants was significantly ameliorated by the treatment with 200 mg/kg DOFE (all, *p* < 0.01). These data indicated that DOFE improved antioxidant capacity, reduced lipid peroxidation, and promoted antioxidant defense.

### 3.4. Effect of DOFE on the Nuclear Factor Erythroid 2-Related Factor 2 (Nrf2)/Heme Oxygenase-1 (HO-1)-Mediated Antioxidant Response

**Figure 4:**
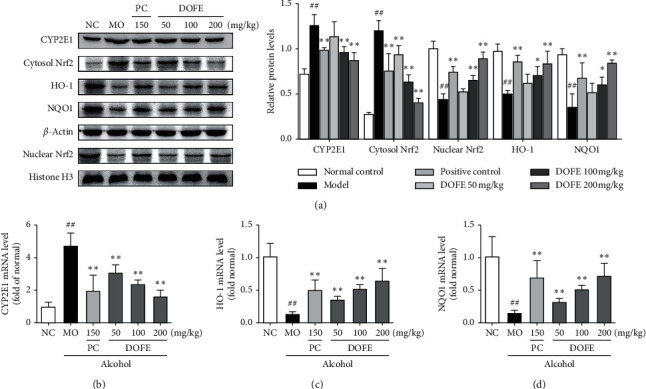
Effect of DOFE on Nrf2/HO-1-mediated antioxidant response. (a) The expressions of the CYP2E1, Cytosol Nrf2, HO-1, and nuclear Nrf2 were assessed by Western Blotting (*n* = 3). *β*-actin and Histone H3 were used as a loading control. Relative mRNA expression of (b) CYP2E1, (c) HO-1, and (d) NQO1. All data are expressed as mean ± SD (*n* = 8). #*p* < 0.05, ##*p* < 0.01 vs. normal control group; ^*∗*^*p* < 0.05, ^*∗∗*^*p* < 0.01 vs. model group.

To understand the underlying mechanism of DOFE protecting against alcohol-induced oxidative stress, the protein expressions of CYP2E1 and antioxidant defense-related genes Nrf2, NAD(P)H quinone oxidoreductase l (NQO1), and HO-1 were examined by quantitative real-time polymerase chain reaction (qPCR) and Western Blotting Analysis (Figure 4). The protein expression and gene expression of CYP2E1 in the MO group increased 1.7-fold and 4.7-fold compared with the NC group (both, *p* < 0.01). This elevation of the protein expression was significantly inhibited by 100 mg/kg DOFE and 200 mg/kg DOFE treatments (both, *p* < 0.01). The elevation of the gene expression was significantly inhibited by all DOFE treatments (all, *p* < 0.01). Similarly, the protein expression of nuclear Nrf2 in the MO group dramatically increased 4.4-fold when compared with the NC group (*p* < 0.01). Conversely, alcohol remarkably decreased the protein expressions of cytosol Nrf2, HO-1, and NQO1 by 56.1%, 48.1%, and 62.0%, respectively, compared with the NC group (all, *p* < 0.01). The depletion of these proteins was significantly reversed by the treatments of 200 mg/kg DOFE (all, *p* < 0.01). In addition, the gene expressions of HO-1 and NQO1 in the MO group dramatically decreased by 88.1% and 86.1% when compared with the NC group (both, *p* < 0.01). These results clearly showed that the protective effects of DOFE against alcohol-induced liver injury are associated with upregulating the expression of CYP2E1 and antioxidant defense-related Nrf2, HO-1, and NQO1 genes.

### 3.5. Effect of DOFE on Alcohol-Induced Inflammation

**Figure 5:**
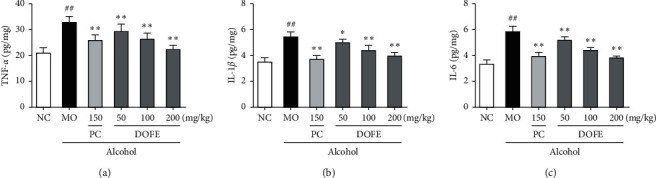
Effect of DOFE on alcohol-induced inflammation. (a) TNF-*α*; (b) IL-1*β*; (c) IL-6. All data are expressed as mean ± SD (*n* = 8). #*p* < 0.05 and ##*p* < 0.01 vs. normal control group; ^*∗*^*p* < 0.05 and ^*∗∗*^*p* < 0.01 vs. model group.

To evaluate the effect of DOFE on alcohol-induced inflammation, the expressions of TNF−*α*, IL-1*β*, and IL-6 were determined (Figure 5). The levels of these cytokines were significantly higher in the MO group than those in the NC group. The results indicated that the liver suffered the alcohol-induced inflammatory response (all, *p* < 0.01). Promisingly, DOFE significantly reduced the levels of TNF-*α*, IL-1*β*, and Interleukin-6 (IL-6) in the liver compared with the MO group (all, *p* < 0.01). These results suggested that DOFE might improve alcohol-induced liver injury via suppressing the inflammatory response.

**Figure 6:**
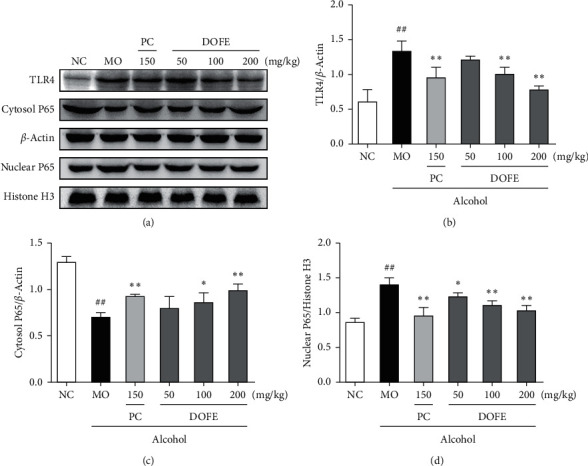
Effect of DOFE on TLR4-mediated inflammatory response. (a) The expression levels of the TLR4, cytosol NF-*κ*B P65, and nuclear NF-*κ*B P65. Quantitative results of Western Blotting analyses for (b) TLR4, (c) cytosol NF-*κ*B P65, and (d) nuclear NF-*κ*B P65. *β*-actin and Histone H3 were used as a loading control. All data are expressed as mean ± SD (*n* = 3). #*p* < 0.05 and ##*p* < 0.01 vs. normal control group; ^*∗*^*p* < 0.05 and ^*∗∗*^*p* < 0.01 vs. model group.

To examine the effect of DOFE on the alcohol-induced inflammatory response, the expressions of proteins related to the TLR4-mediated signaling pathway were examined (Figure 6). Alcohol caused a 2.2-fold increase in the protein expression of TLR4 compared with NC mice (*p* < 0.01). DOFE (100 mg/kg and 200 mg/kg) treatment significantly inhibited this alcohol-induced inflammation (both, *p* < 0.01). Compared with the NC group, alcohol exposure also decreased the protein expression of its downstream NF-*κ*B P65 by 46.5% and enhanced the nuclear NF-*κ*B P65 by 1.6-fold (both, *p* < 0.01), which were normalized by bifendate and 200 mg/kg DOFE treatments (all, *p* < 0.01). The results revealed that DOFE reversed the upregulation of hepatic TLR4 and nuclear NF-*κ*B P65 protein expression and the increase in the level of the inflammatory factor in the liver caused by alcohol. All these results demonstrated that DOFE might affect TLR4-mediated inflammatory response induced by alcohol exposure to reverse the alcohol-induced liver injury.

## 4. Discussion

Alcohol-induced liver injury is a common liver injury with a single and clear causative factor, the alcohol accumulation [11, 28]. When the liver is stimulated by alcohol, hepatocyte inflammation, necrosis, and steatosis can occur. Acetaldehyde, one of the alcoholic metabolites, can not only damage liver tissues directly but also destroy mitochondria and microtubules of most liver cells, resulting in excessive oxidation of unsaturated fatty acids, dysfunction of protein secretion, and oil accumulation [16, 29]. Meanwhile, excessive ethanol accumulation also can affect fat metabolism, resulting in fat accumulation in liver cells. In addition, alcohol metabolism produces a great number of reactive oxygen radicals, which lead to excessive oxidation of lipids [28, 29].

The serum ALT and AST levels are the most sensitive indicators of the liver damage and indirectly reflect the extent of the liver damage [30]. The increasing serum of ALT and AST would indicate liver cell damage or necrosis [31]. Our data suggested that the serum ALT and AST levels induced by alcohol in the DOFE group could be effectively downregulated. It is demonstrated that DOFE showed a good protective effect on alcohol-induced liver injury. Another important manifestation of alcoholic liver injury is lipid metabolism disorder, which is mainly manifested by the deposition of TG in the liver [28]. Previous studies have shown that the serum levels of TC and TG increased when the mice are given alcohol by gavage [32]. Our results also showed that alcohol exposure in mice could lead to the disorderliness of lipid metabolism and the increase of serum TC and TG levels. At the same time, DOFE could significantly reduce abnormal lipid levels, effectively decreasing the lipid accumulation caused by alcohol.

Alcohol metabolism produces many reactive oxygen radicals, and oxidative stress is an essential mechanism of alcoholic liver injury [33–35]. In the pathogenesis of alcohol-induced liver injury, oxidative stress plays a crucial role [36]. MDA, the end-product of excessive oxidation of lipids, is a general index of oxidative damage [37]. As an enzyme for catalyzing the conversion of superoxide anions to hydrogen peroxide and oxygen, SOD could maintain cellular redox balance and scavenge ROS [38]. GSH has been recognized as an important substrate in GSH-related antioxidation and detoxification [39]. GSH-Px specifically catalyzes the reduction reaction of GSH to peroxides to block the chain reaction of lipid peroxidation [39]. Therefore, increasing those enzymatic and nonenzymatic antioxidants may be conducive to relieve alcohol-induced oxidative stress [5, 40]. The results of this study demonstrated that DOFE could reduce the level of MDA and increase the activity of SOD, CAT, and GSH-PX activities and the GSH level. The hepatoprotective activity of DOFE could be returned to its contents of polyphenol, flavones, and alkaloids with their antioxidant characteristics. Studies indicated that rutin, schaftoside, vitexin, and quercetin could increase the antioxidant status in mice liver, which was consistent with our experimental results [41–43].

The CYP2E1, as one of the main members of this enzyme system, plays a crucial role in the oxidative stress mechanism in the alcoholic liver. The production of CYP2E1 will aggravate oxidative stress [44, 45]. Alcohol is a strong inducer of CYP2El [46]. In our study, the increased expression CYP2E1 gene and protein were significantly attenuated by the treatment of DOFE. To explore the mechanism of DOFE alleviating alcohol-induced liver injury, the expression of Nrf2 and its downstream protein HO-1 and NQO1 were also examined by Western Blotting and qPCR. Nrf2, a transcription factor, can induce many encoding detoxification enzymes of downstream genes and antioxidant proteins to relieve oxidative stress. Recently, Nrf2 has been recognized as a new target for the treatment of alcohol-induced liver injury [47–50]. Our results demonstrated that DOFE treatment inhibited the alcohol-induced elevation of cytosol Nrf2, HO-1, and NQO1 expressions. These results suggest that the protective effects of DOFE against alcohol-induced liver injury might reduce oxidative stress through the downregulation of CYP2E1 expression and the activation of the Nrf2/HO-1 pathway.

Evidence shows that inflammation is an essential factor which contributes to the initiation and progression of alcoholic liver disease [51]. Toll-like receptors (TLRs) have some protective effects when pathogens invade the body and activate signaling pathways that induce immune and proinflammatory gene expression [52]. Researches have shown that TLR4 plays a crucial role in many therapeutic targets for inflammation-related diseases [53]. Besides, previous studies have shown that mutant TLR4 mice [54], or mice deficient in TLR4 [22, 55], display a lower probability of alcohol-induced fatty liver disease. The activation of TLR4 can also upregulate levels of TNF-*α*, IL-1*β*, and IL-6 [56]. NF-*κ*B P65 is activated by stimuli or cellular stress, such as TLRs, TNF-*α*, andIL-1*β* [57]. In this study, our results indicated that alcohol exposure increased the protein expressions of TLR4, nuclear NF-*κ*B P65, and the levels of TNF-*α*, IL-1*β,* and IL-6. On the contrary, these increases were suppressed by DOFE. All these results demonstrated that DOFE inhibits TLR4/NF-*κ*B-mediated inflammatory response induced by alcohol exposure.

## 5. Conclusions

**Figure 7:**
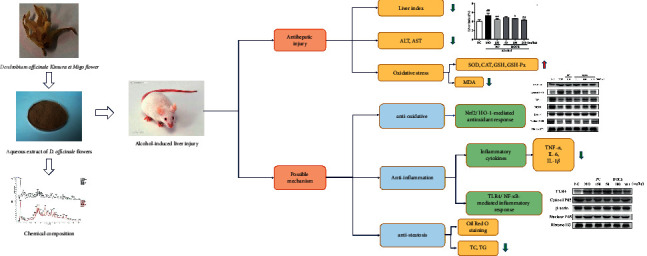
Graphical Abstract. *Dendrobium officinale* flower extraction mitigates alcohol-induced liver injury in mice: the role of antisteatosis, antioxidative, and anti-inflammatory mechanisms.

Treatment with DOFE effectively protects against alcohol-induced liver injury. This protective effect might be associated with inhibiting steatosis, activating Nrf2/HO-1-mediated antioxidant defense, and suppressing TLR4/NF-*κ*B -mediated inflammation. These findings also suggest that DOFE is a functional food for protecting against alcohol-induced liver injury (Figure 7).

## Figures and Tables

**Table 1:** Primer sequences.

Targeted gene	Direction and sequence (5′ to 3′)
CYP2E1	Forward: GGAAGGACGTGCGGAGGTTTTC
	Reverse: TCCACCAGGAAGTGTGCCTCTC
HO-1	Forward: ACCGCCTTCCTGCTCAACATTG
	Reverse: CCTCTGACGAAGTGACGCCATC
NQO1	Forward: ATCCTGCGTTTCTGTGGCTTCC
	Reverse: TCCTCCCAGACGGTTTCCAGAC
GAPDH	Forward: GGTTGTCTCCTGCGACTTCA
	Reverse: TGGTCCAGGGTTTCTTACTCC

**Table 2:** Identification of the chemical constituents in DOFE.

Number	Retention time (min)	Ion mode	Extraction mass (Da)	Found mass (Da)	Error (ppm)	Formula	Identification	Peak area (%)
1	1.57	+	169.0739	169.0739	0.1183	C_8_H_11_NO_3_	Pyridoxine	10.0524
2	2.83	+	244.0692	244.0695	1.5160	C_9_H_12_N_2_O_6_	Uridine	0.0371
3	7.73	−	180.0424	180.0423	−0.6665	C_9_H_8_O_4_	Caffeic acid	0.4826
4	8.05	−	564.1464	564.1479	2.7298	C_26_H_28_O_14_	Schaftoside	4.6005
5	8.66	+	610.1513	610.1534	3.3926	C_27_H_30_O_16_	Rutin	3.6911
6	8.76	−	432.1048	432.1057	1.9440	C_21_H_20_O_10_	Vitexin	0.8948
7	8.92	+	464.0943	464.0955	2.4779	C_21_H_20_O_12_	Quercetin-3*β*-D-glucoside	2.0444
8	8.92	−	302.0418	302.0427	2.9135	C_15_H_10_O_7_	Quercetin	0.6892
9	9.52	+	316.0576	316.0583	2.3097	C_16_H_12_O_7_	Isorhamnetin	0.2671
10	11.92	−	330.2396	330.2406	3.0584	C_18_H_34_O_5_	(15Z)-9,12,13-Trihydroxy-15-octadecenoic acid	1.2793
11	14.73	−	314.2449	314.2457	2.5776	C_18_H_34_O_4_	(±) 12 (13)-DiHOME	3.7790
12	16.61	+	294.2188	294.2195	2.2092	C_18_H_30_O_3_	9-Oxo-10 (E), 12 (E)-octadecadienoic acid	2.4793
13	19.58	+	282.2797	282.2785	−4.1094	C_18_H_35_NO	Oleamide	1.5290
14	23.34	+	338.3423	338.3413	−2.8078	C_22_H_43_NO	Erucamide	2.2491

## Data Availability

The data used to support the findings of this study are available from the corresponding author upon request.
